# Resilience in Adolescent Girls in Child Welfare: Reliability and Validity of the RS-14

**DOI:** 10.1007/s10560-023-00952-x

**Published:** 2023-11-28

**Authors:** Wendy Auslander, Shih-Ying Cheng, Tonya E. Edmond

**Affiliations:** 1 George Warren Brown School of Social Work, Washington University in St. Louis, 1 Brookings Drive, Campus, Box 1196, St. Louis, MO 63130 USA; 2 University of Illinois Chicago, Chicago, USA

**Keywords:** Resilience, Adolescents, Child Welfare, RS-14, Validity, Reliability

## Abstract

Adverse childhood experiences, such as abuse and neglect, have been shown to have longstanding negative consequences on a child’s development and outcomes. Studies have noted that there is variation in how youth in child welfare respond to adversity, yet few studies have examined the psychometrics of measures of resilience in this population. In particular, the 14-item Resilience Scale (RS-14) is a widely used instrument yet has not been evaluated for use with adolescents in child welfare populations. The purpose of the study was to describe the levels of resilience reported by adolescent girls involved in the child welfare system and to evaluate the reliability, validity, and factor structure of this scale in this population. Participants were 249 adolescent girls, ages 12–19, who were involved in the child welfare system. Interviews assessed resilience, symptoms of post-traumatic stress, depression, social problem-solving, and demographic variables. Results indicated that levels of resilience among the participants were in the moderate range. The RS-14 demonstrated evidence of good internal consistency and test–retest reliability. Convergent and discriminant validity were established. Confirmatory factor analysis testing a single-factor solution resulted in a weak model fit. A follow-up exploratory factor analysis supported a two-factor solution. Findings suggest this instrument is an appropriate tool for use in child welfare populations.

Adverse childhood experiences, such as histories of childhood abuse and neglect, have consistently been shown to be associated with negative consequences, such as mental and physical health problems, revictimization, aggression, substance abuse, and risk behaviors in adolescence and adulthood (Auslander et al., 2002, 2018; Felitti et al., 1998). Child maltreatment is relatively common in the U.S. with 37.4% of all children experiencing a formal investigation by child protective services by the time they are age 18 (Kim et al., 2017). Despite the increased risk of negative outcomes in this population, studies have noted that there has been much variability in how children respond to adversity and stress. Children who have coped effectively with significant adversity were more likely to demonstrate adaptive outcomes, such as social competency, academic success, and positive behavioral adjustment (Horn et al., 2016; Masten & Barnes, 2018).

Resilience has been broadly defined as positive adaptation in the context of significant adversity (Masten & Barnes, 2018; Rutter, 1987) and operationalized in two major ways: a personal characteristic that contributes to an outcome, or the actual outcome itself (Kaplan, 1999; Luthar et al., 2000). Personal characteristics have been conceptualized as a set of qualities or traits such as self-esteem, self-efficacy, competence, problem-solving ability, and autonomy that can moderate the negative effects of adversity and enable positive adaptation (Rutter, 1987). The second and more common way that resilience has been operationalized is the presence or absence of positive or negative outcomes in response to adversity. Common outcomes that have been used to indicate resilience among youth who have experienced child maltreatment include measures of positive functioning, developmental achievements, and mental health (Domhardt et al., 2015; Yoon et al., 2021). Researchers have suggested that youth with histories of child abuse and neglect may demonstrate resiliency in one area of functioning (i.e., academic achievement) and not in another (i.e., mental health; Domhardt et al., 2015). Because of this many studies have utilized multidimensional instruments to assess multiple areas of mental health and behavioral competence to measure resilience in this population (Bell et al., 2013; Edmond et al., 2006; Jones, 2012). However, few studies with adolescents in child welfare have operationalized resilience using a specific measure of individual characteristics that enable youth to adapt successfully to adversity. One such measure, the RS-14 Resilience Scale (RS-14; Wagnild & Young, 1993) operationalized resilience as intrapersonal characteristics using a shortened version of the original Resilience Scale.

The original Resilience Scale was comprised of 25 items that were developed through qualitative interviews of adult women who successfully recovered from a major life event. Since its development, the Resilience Scale has demonstrated good reliability and validity in many studies and has been used with individuals from varied ethnic backgrounds and ages worldwide (Wagnild, 2011). More recently, in order to decrease respondent burden, the RS-14 version of the original scale was developed (Wagnild, 2011) which consisted of 14 items using a 7-point scale ranging from “1 = Strongly Disagree” to “7 = Strongly Agree” with higher total scale scores indicating higher levels of resilience.

A growing number of studies have evaluated the psychometric properties of the RS-14. One systematic review of these studies noted that the instrument has been translated into ten languages with good cross-racial validity and reliability (Miroševič et al., 2019). However, the majority of the psychometric investigations of the RS-14 were conducted with adult samples ages 18 years and older. Among those few studies that included adolescent samples, only one study to date has examined the psychometric properties of the RS-14 utilizing a sample of adolescents in the U.S. (Pritzker & Minter, 2014). The study included a large school-based sample of 2,982 adolescents, who were ethnically and economically diverse. Results indicated that the instrument demonstrated excellent internal consistency reliability and validity, with a single-factor structure (Pritzker & Minter, 2014). Moreover, it was noted that the RS-14 was brief and easy to administer and score with adolescents.

Despite the critical need to better understand and assess resilience among youth with histories of adverse childhood experiences such as abuse and neglect, there have been no studies to date that have examined the psychometric properties of the RS-14 among adolescents in the child welfare system. Moreover, because adolescents involved in child welfare can be a diverse population, examining the appropriateness of the RS-14 for those in need of mental health services may be particularly important in order to assess their levels of resilience. To address this important gap in knowledge, the present study investigated the psychometric properties of the RS-14 with a sample of adolescent girls who were currently involved in the U.S. child welfare system and who were referred to a trauma treatment intervention. Specifically, the following questions were addressed in this study:

1. What is the level of resilience reported by adolescent girls involved in the child welfare system as defined by scores on the RS-14, and are there differences by demographics (i.e., age, race, current living situation)?
2. To what extent does the RS-14 demonstrate reliability and validity among child welfare-involved adolescent girls with histories of abuse and neglect?
3. What is the factor solution or dimensionality of the RS-14 among child welfare adolescents?

## Method

### Participants

Participants were 249 girls, ages 12–19 (*M* = 14.9, *SD* = 1.6) involved in the state’s child welfare service agency located in a metropolitan area in the Midwest. The girls were recruited for a group trauma-focused cognitive-behavioral intervention study due to previous experiences of significant trauma and related emotional or behavioral problems. The majority of analyses in this study combined pretest data from participants in both the intervention condition and control condition were used, resulting in a cross-sectional study design. To determine the test–retest reliability of the RS-14, the pretest and posttest data from the control group were used, eliminating any possible impact of the intervention on the results.

Eligible participants were adolescent girls who were (a) ages 12–19 years old and (b) currently involved in the child welfare system (i.e., those who were formally investigated and substantiated for child maltreatment by child protective services). Girls were excluded if they met any of the following criteria: (a) were unable to read or write, (b) lived in the same residence as another study participant, (c) were unable to tolerate discussing abuse or neglect, or (d) if they had behaviors that would prohibit participation in a group treatment or interview.

As shown in Table 1, the majority of participants were African American (*n* = 173, 69.5%), followed by White, non-Hispanic (*n* = 63, 25.3%). About nine out of ten participants (*n* = 220, 88.4%) lived in non-congregate care which included biological parents, relatives, adoptive parents, and foster homes, and the remainder (11.65%, *n* = 29) lived in congregate care (i.e., group homes and residential treatment facilities). Additionally, about three out of five adolescent girls lived with foster families, relative care, adoptive care, and other types of group homes (60.6%, *n* = 151), and two-fifths (39.4%, *n* = 98) lived with their biological parents.

**Table 1:** Sample characteristics

Variable	*n* (%) or *M (SD)*
Race	
White not Hispanic	63 (25.30)
Youths of color^a^	186 (74.70)
Current living situation	
Biological parent	98 (39.36)
Other	151 (60.64)
Congregate care	
Yes	29 (11.65)
No	220 (88.35)
Resilience scores	
Low (< 65)	47 (18.88)
Moderately low to moderate (65–81)	103 (41.37)
Moderately high (82–90)	62 (24.90)
High (> 90)	37 (14.86)
Age (years)	14.89 (1.63)
Resilience total score	75.88 (13.85)
PTSD total score	16.77 (10.99)
Depression total score	11.98 (8.48)
Social problem-solving total score	11.49 (2.64)

*N* = 249. Sample size varies due to missing data

^a^Of the sample, 69.5% (*n* = 173) were African American, 1.6% (*n* = 4) Hispanic, 0.4% (*n* = 1) Asian, 1.6% (*n* = 4) American Indian, and 1.6% (*n* = 4) Other

### Procedures

The study was approved by the Human Subjects Institutional Review Board of the two collaborating universities, Washington University in St. Louis and the University of Missouri-St. Louis, and the state child protective services agency (i.e., Children’s Division of Missouri). Additionally, a Certificate of Confidentiality was obtained by the Centers for Disease Control, the study’s funding agency. The recruitment and informed consent procedures included several steps. Participants were recruited through referrals from case managers or other members of the family support team of Children’s Division. After the adolescent expressed interest in participating in the study to the case manager or family support team member, the referral was provided to the study coordinator who then reached out to the adolescent girl to explain the details of the study. Written consent was then obtained from the legal custodian, which could be the biological parent, relative, adoptive parent, or the youth’s case manager. Last, written consent of members of the family support team (e.g., guardian ad litem, juvenile officer, and child’s current therapist) was secured. All adolescents under the age of 18 provided written assent to participate in the study, and those youth ages 18 or older provided consent. Each of the adolescent girls received a $20 gift card for participating in the study.

Data for this psychometric evaluation of the RS-14 were collected using pen and paper interviews that were administered over 3.5 years between 2007 and 2011 by master’s and doctoral-level social work students. All interviewers participated in eight hours of interview training that included background knowledge of the population, basic research interviewing skills, confidentiality and ensuring privacy during the interview, and procedures for reports of suicide-related items and abuse. The study interviews were conducted in private space either in a community-based mental health agency, the Children’s Advocacy Services of Greater St. Louis, or in the participant’s residence. Interviews lasted approximately one hour to complete.

## Variables and Measures

### Resilience

The 14-item Resilience Scale (RS-14; Wagnild, 2011) was used to assess resilience characteristics. Items explored participants’ perceptions of statements that describe their ability and positive beliefs about themselves to cope with difficult situations. Participants rated each item using a seven-point Likert scale from 1 = “Strongly disagree” to 7 = “Strongly agree.” The possible total scale scores ranged from 14–98, with higher scores indicating higher resilience. The developers of the RS-14 suggested categories of total scale scores to interpret the degree of “low” resilience (14–64), “moderately low to moderate” resilience (65–81), “moderately high” resilience (82–90), and “high” resilience (91–98; see Wagnild, 2011).

### PTSD Symptoms

Posttraumatic stress symptoms were assessed using the Child PTSD Symptom Scale (CPSS; Foa et al., 2001). Seventeen items (e.g., Having bad dreams or nightmares; Trying not to think about, talk about, or have feelings about the trauma) assessed domains of re-experiencing, avoidance, and arousal. Participants rated the frequency of posttraumatic symptoms over the past month using a 4-point scale from 0 = “Not at all” to 3 = “Five or more times a week.” A total scale score of ≥ 15 was used as a clinical cutoff for this study as recommended by the International Society for Traumatic Stress Studies (n.d.). The CPSS has previously demonstrated convergent validity, correlating highly with a similar PTSD scale (Foa et al., 2001). Good internal consistency and test–retest reliability have also been established (Foa et al., 2001). In the present study, internal consistency reliability was strong (α = 0.90).

### Depression

The Child Depression Inventory (CDI; Kovacs, 2003) was used to measure depressive symptoms over the previous 2 weeks. The 27 items were rated from 0 to 2 and summed, with higher scores indicating more severe symptoms. A clinical cut-off score of ≥ 13 has been proposed for use with clinical samples (Kovacs, 2003). Concurrent, discriminative, and criterion validity have been demonstrated (Kovacs, 2003). Good internal consistency and test–retest reliability have been demonstrated for a child welfare population (Kolko et al., 2010). The scale demonstrated good internal consistency reliability for the current sample (α = 0.89).

### Social Problem-Solving

Social problem-solving skills were assessed by the Social Problem-Solving Inventory-Revised: Short Form (D’Zurilla et al., 2002) which measures the cognitive-behavioral processes used by individuals to adapt, cope, and resolve everyday problems. The scale consisted of 25 items that were rated on a 5-point scale that ranged from 0 = “Not at all true of me” to 4 = “Extremely true of me,” with higher scale scores indicating more effective problem-solving. The Social Problem-Solving Inventory-Revised: Short Form has been shown to be reliable and valid among adolescent populations (D’Zurilla et al., 2002). The internal consistency reliability for the current sample was good (α = 0.82).

### Data Analysis

The data were double entry verified and then were imported into SAS 9.4 for final quality checks and analysis. Data analysis involved describing the levels of resilience reported by the participants, and examination of the reliability, validity, and factor structure of the RS-14 scale. Internal consistency reliability of the RS-14 was determined by computing the Cronbach’s alpha coefficient with the total sample. To determine the test–retest reliability of the RS-14, data collected at baseline and at 3 months from a subsample of girls who were in the control care condition (*n* = 108) were utilized. Analyses to compute the test–retest reliability were conducted using both a Pearson correlation coefficient and an Interclass Correlation Coefficient (ICC) using a mixed model (time periods fixed, respondents random) and requiring absolute agreement. Two types of validity were analyzed: convergent and discriminant validity. Convergent validity was examined by computing the correlation between resilience (RS-14) and social problem-solving (SPSI). Because social problem-solving has been considered an indicator or correlate of resilience in childhood (Horn et al., 2016), it was hypothesized that participants who had higher levels of resilience would have greater social problem-solving skills. Second, discriminant validity was examined using *t*-tests to determine if there were significant differences in resilience between girls who scored within the normal versus clinical range for PTSD or depression. Based on previous research that indicated resilience was inversely related to PTSD symptoms (Day & Kearney, 2016), and likewise, resilience was inversely related to depression (Wagnild, 2011), it was hypothesized that girls experiencing fewer PTSD symptoms (< 15) and lower depressive symptoms (< 13) would demonstrate higher levels of resilience. Last, the dimensionality of the RS-14 was investigated using confirmatory factor analysis (CFA) followed by exploratory factor analysis (EFA). For EFA, we used maximum likelihood factor analysis with subsequent VARIMAX rotation.

## Results

Results of the descriptive analyses to determine the levels of resilience among the adolescent girls in the study indicated that the mean total score of the RS-14 was 75.88 (*SD* = 13.85), with a range of 28 to 98. The mean score was within the range that Wagnild (2011) defined as moderate resilience (i.e., total mean scores falling between 74 and 81; Wagnild, 2011). As shown in Table 1, the frequency of girls by degree of resilience were as follows: low (*n* = 47, 18.88%), moderately low to moderate (*n* = 103, 41.37%), moderately high (*n* = 62, 24.90%), and high (*n* = 37, 14.86%). Table 2 presents the means and standard deviations of each item. Table 3 presents the numbers and percentages of girls who responded in the most positive categories or top two categories (i.e., 6 or 7) on the seven-point scale. Results indicated that the most highly endorsed items were as follows: feelings of pride that they have accomplished things in life (73.49%), finding something to laugh about (73.49%), and believing their life has meaning (72.29%). The least endorsed items among the girls were handling many things at a time (35.75%), taking things in stride (37.35%), and having self-discipline (40.57%). Further analyses were conducted to determine if there were differences in the total resilience scores according to the participants’ age, race (youth of color vs. White), and current living situation (biological parent vs non-biological parent). Results of these analyses indicated that there were no significant associations between age and resilience (*r* = 0.09, *p* = 0.16). Similarly, no significant differences were found in levels of resilience according to race (*t*(247) = 1.23, *p* = 0.22) or current living situation (*t*(247) = 1.44, *p* = 0.15).

**Table 2:** Descriptive statistics by Item on RS-14

Item	Description	*M*	*SD*
1	Manage one way or another	5.21	1.68
2	Proud that I have accomplished things in life	6.10	1.32
3	Take things in stride	4.77	1.80
4	Friends with myself	5.56	2.04
5	Handle many things at a time	4.78	1.74
6	Determined	5.65	1.59
7	Get through difficult times because experienced difficulty before	5.65	1.60
8	Self-discipline	4.73	1.92
9	Interested in things	5.19	1.68
10	Find something to laugh about	6.02	1.29
11	Belief in myself gets me through hard times	5.63	1.66
12	I’m someone people generally rely on	5.35	1.69
13	Life has meaning	5.86	1.67
14	In a difficult situation, can find my way out	5.38	1.72
Total scale		75.88	13.85

*N* = 249

**Table 3:** Percentage of participants with high levels of agreement ^a^ by item on RS-14

Item	Description	*n*	%
1	Manage one way or another	122	49.00
2	Proud that I have accomplished things in life	183	73.49
3	Take things in stride	93	37.35
4	Friends with myself	165	66.26
5	Handle many things at a time	89	35.75
6	Determined	162	65.06
7	Get through difficult times because experienced difficulty before	155	62.25
8	Self-discipline	101	40.57
9	Interested in things	119	47.79
10	Find something to laugh about	183	73.49
11	Belief in myself gets me through hard times	164	65.87
12	I’m someone people generally rely on	137	55.02
13	Life has meaning	180	72.29
14	In a difficult situation, can find my way out	141	56.63

*N* = 249

^*a*^Participants who endorsed the strongest level of agreement (6 or 7) for items on the RS-14

Results of the internal consistency reliability analyses indicated that the RS-14 demonstrated high reliability (α = 0.85) in this sample. Data for the test–retest reliability analyses were collected at baseline and at 3 months from the subsample of girls in the control group condition. There were no significant differences at baseline in the resilience scores for the girls in the control group and experimental conditions (*t*(232) = − 0.23, *p* = 0.81), suggesting that the participants in the control group condition were representative of the total study sample in their resilience levels. Test–retest reliability of the RS-14 was determined using both the Pearson Correlation Coefficient (*r* = 0.74, *p* < 0.0001) and the single rater ICC using absolute agreement criterion (ICC = 0.73, *p* < 0.001, 95% confidence interval 0.63–0.81). Both of these stability coefficients indicated acceptable test retest-reliability. Convergent validity was tested by examining the relationship between resilience and social problem-solving skills. As expected, results indicated that girls who reported higher levels of resilience had more effective social problem-solving skills than those with lower levels of resilience (*r* = 0.47*, p* < 0.0001*, n* = 248). Discriminant validity was assessed by comparing the RS-14 scores for participants scoring above and below the clinical cutoff scores for PTSD and depression. Results confirmed our hypotheses that the RS-14 differentiated between girls who scored above and below the clinical cut-off scores for PTSD (*t*(246) = 5.15,* p* < 0.0001]; girls with higher levels of resilience reported significantly lower levels of PTSD (*M* = 71.94, *SD* = 14.01) compared to their counterparts with lower resilience levels (*M* = 80.58, *SD* = 12.12). Likewise, the RS-14 differentiated between girls with above and below the clinical cut-off scores for depression (*t*(244) = 6.42, *p* < 0.0001]; girls with higher levels of resilience reported lower levels of depression (*M* = 69.34, *SD* = 14.43) compared to those with lower levels of resilience (*M* = 80.11, *SD* = 11.77).

Last, to investigate the dimensionality of the RS-14, CFA was conducted to test the fit of a single dimension solution suggested in two previous studies with adolescents and adults (Pritzker & Mintner 2014; Wagnild, 2011). The results of the CFA indicated that although all factor loadings were significant and in the expected direction, the model fit was relatively weak with a significant model chi square *χ*^2^ = 239.241, *df* = 77, *p* <  0.0001), low AGFI (0.808), and high RMSEA (0.092; Schreiber et al., 2006). Because of the relatively weak fit with a single-factor solution, a follow-up maximum likelihood exploratory factor analysis (EFA) with squared multiple correlations used for communality estimates was conducted to determine whether a meaningful multiple dimension model would emerge. The number of factors retained in the EFA was determined using criteria described by Gorsuch (1983): (a) minimum eigenvalue of 1, (b) no factor having less than three items, and (c) the solution resulting in meaningful and easily interpretable dimensions. Based on these criteria, a two-factor solution was chosen, with Factor I representing “Belief in Oneself” (7 items) and Factor II representing “Personal Competence” (7 items). The total variance explained by Factor I and Factor II were 61% and 12% respectively. The factor loadings based on a VARIMAX rotation are presented in Table 4. The alpha coefficients for subscales based on Factors I and II were α = 0.82 and α = 0.76, respectively.

**Table 4:** Results of exploratory factor analyses

Item	Description	Factor	
		I	II
11	Belief in myself gets me through hard times	0.79	0.19
13	Life has meaning	0.79	0.22
2	Proud that I have accomplished things in life	0.58	0.24
10	Find something to laugh about	0.56	0.12
9	Interested in things	0.56	0.31
4	Friends with myself	0.54	0.27
12	I’m someone people generally rely on	0.34	0.24
7	Get through difficult times because experienced difficulty before	0.12	0.62
5	Handle many things at a time	0.19	0.60
6	Determined	0.33	0.59
14	In a difficult situation, can find my way out	0.30	0.48
1	Manage one way or another	0.12	0.48
8	Self-discipline	0.20	0.46
3	Take things in stride	0.31	0.37

*N* = 249

Factor I: belief in oneself; Factor II: personal competence

## Discussion

Descriptive findings of the current study indicate that the levels of resilience as measured by the RS-14 (*M* = 75.88) fall within the moderate range as defined by the scale developer (Wagnild, 2011). To date, there have been no previous studies that have used this instrument with adolescents currently involved in the child welfare system. However, results from the present study are very consistent with the levels of resilience measured by the RS-14 in several studies with ethnically diverse adolescents, such as Pritzker and Mintner’s (2014) school-based study in the U.S. (*M* = 79.3), pregnant adolescents in Ecuador (*M* = 79.30; Salazar-Pousada et al., 2010), Lithuanian adolescents (*M* = 70.48; Surzykiewicz et al., 2019) and aboriginal adolescents in India (*M* = 73.65; Ritchie et al., 2014). It is not surprising that the majority of girls in the current study demonstrated moderate levels of resilience; there is strong evidence that adults with histories childhood abuse often experience positive behavioral, mental health, and health outcomes despite their childhood adversity (DuMont et al., 2007). Additionally, because these youth were involved in the child welfare system, many of them may have had some prior exposure to mental health services before they were referred to the present study. Consistent evidence has shown that child welfare youth receive mental health services more frequently than other adolescent populations (McMillen et al., 2004), and it is possible that these services may have helped them to build resilience over time. Evidence has shown that many mental health interventions for children and youth in the child welfare system have proven to be effective (Craven & Lee, 2006).

It should be noted that despite the moderate range of resilience scores among the majority of girls in this study and the skewed responses to the higher end, 18.9% (*n* = 47) of the girls reported “very low” or “low” levels of resilience. This finding suggests that approximately one-fifth of the participants may be at high risk of experiencing the negative consequences of childhood abuse and neglect and of high need for services and interventions to increase their ability to “cope and bounce back” from their adversity.

A systematic review of resilience in sexually abused children and youth found rates of resilience that ranged from 10 to 53% (Domhardt et al., 2015). The wide variation in rates of resilience may be explained by the number of different ways that resilience has been operationalized in the literature. Many previous studies have conceptualized resilience as the absence of negative outcomes such as depression, hopelessness, anxiety, or the presence of positive outcomes such as academic success, and positive behavioral adjustment, in addition to personal characteristics as measured by the RS-14.

One of the current study’s important findings was that the RS-14 demonstrated high internal consistency reliability in a sample of child welfare-involved adolescent girls. In addition, results provided evidence for the test–retest reliability or stability of the RS-14, which had not been established previously in any adolescent population. Last, evidence for the validity of the instrument was established. Analyses indicated that the scale showed significant positive correlations with a measure of social problem-solving, which is considered an indicator or correlate of resilience in youth (Horn et al., 2016). Additionally, the RS-14 was able to differentiate girls who reported symptoms of PTSD and depression in the clinical range from their non-clinical counterparts, providing evidence for the instrument’s discriminant validity in this population. Thus, the RS-14 was determined to be an appropriate instrument for assessing resilience in adolescent girls with histories of child maltreatment.

In the current study, two underlying dimensions of resilience emerged for this population: Factor I: “Belief in Oneself” and Factor II: “Personal Competency.” These results differ from the one-factor solution described by the developer of the scale (Wagnild, 2011) in a large adult sample, as well as the one-factor solution in school-based adolescents in the U.S. (Pritzker and Mintner, 2014). Interestingly, the two dimensions identified in the current study are similar to the two-factor structure that was found in the original 25-item scale (Wagnild, 2011). Moreover, the two-factor structure is theoretically meaningful in that it is consistent with previous theory and conceptualizations of resilience as well as a shortlist of protective factors in child development literature (Masten & Barnes, 2018; Rutter, 1987). As discussed by Masten and Barnes (2018), personal characteristics of resilience such as a positive view of oneself and a belief that life has meaning and purpose aligns with Factor I: Belief in Oneself, and active coping and problem-solving aligns with Factor II: Personal Competence.

Additionally, results from the factor analysis indicated that the most highly endorsed items by participants loaded on the first factor “Belief in Oneself,” rather than on the second factor “Personal Competence.” These findings suggest that the characteristics of resilience that most strongly resonated among the girls related to their acceptance of self and positive self-esteem, endorsing feelings of pride for their accomplishments and self-determination. The second factor of resilience (i.e., Personal Competence), consisting of items related to coping, managing, and handling difficult circumstances were not as highly endorsed, suggesting that the girls’ perceptions of their capabilities and skills in this area may warrant some attention to enhance resilience in this population. However, because these two potential dimensions are based on an EFA, further testing of the dimensionality of the RS-14 with a different sample of child welfare-involved youth using CFA is needed.

The current study findings should be considered in light of some limitations. Because the sample was a convenience sample composed of adolescent girls who were referred to a trauma-focused cognitive-behavioral intervention, the sample is not representative of the general child welfare population. Additionally, because resilience has been shown to vary by a myriad of environmental and developmental factors (Yoon et al., 2021), the study results may not represent the level and dimensions of resilience for children and youth involved in the child welfare system who are pre-adolescents or emerging adults. Last, the sample size for the study was small; future research with a larger number of child welfare youth would add to the evidence of the utility and strength of the RS-14 in this population.

The limitations and findings of this study suggest areas for future research. For example, research with a more representative sample of child welfare-involved youth, including males, and ethnically and racially diverse participants would be useful in establishing normative data for this population. Additionally, a larger sample size is warranted. Due to the variations in how resilience has been measured in previous studies, future research with child welfare populations may benefit from consistency across studies in how resilience is assessed. The operationalization of resilience reflected in the items of the RS-14 focuses on youths’ personal characteristics, specifically positive perceptions of themselves and their ability to cope with difficult situations. This may be a useful instrument to use in research examining the buffering or moderating role of resilience in the relationship between child maltreatment and behavioral, mental health, and health outcomes in child welfare adolescents. One such study utilizing the RS-14 indicated that resilience significantly moderated the negative influence of sexual abuse on outcomes such as depression, PTSD and peer revictimization among girls in child welfare (Tlapek et al., 2017). Yet, resilience did not significantly moderate the relationship between physical abuse and these outcomes. In another study with child welfare-involved adolescents, analyses indicated that resilience significantly moderated the relationship between number of types of traumas experienced and PTSD, depression, perpetration, and revictimization; for girls with higher levels of resilience, the negative impact of trauma on mental health problems and peer bullying behaviors was weakened (Auslander et al., 2017). These findings provide some evidence that resilience as measured by the RS-14 may have a protective or buffering effect in this population, yet the effect may vary according to the type and severity of the trauma experienced as well as the outcomes. Future research using the RS-14 is warranted to deepen our understanding of the specific ways in which resilience can impact the physical and psychological well-being of youth with histories of child maltreatment.

Findings from the current study may also have clinical implications for this population. Because findings from this study provided evidence for the test–retest reliability or stability of the RS-14 over a 3-month period, this instrument may be a useful measure in longitudinal designs to evaluate the effectiveness of interventions in child welfare-involved youth. In particular, for resilience-promoting programs, the RS-14 resilience may be conceptualized as an intermediate outcome or pathway (mediator) to distal outcomes such as mental health, behavioral adjustment, and school achievement. However, more research is warranted to assess the extent to which the RS-14 is responsive or sensitive to change, since low sensitivity of an outcome measure can result in a reduction of statistical power in a study of intervention effectiveness (Ching et al., 2015).

In addition to assessing response to treatment, it has also been suggested that this instrument be utilized as a screening tool by clinicians such as family medicine practitioners (Miroševič et al., 2019), or by social workers. Assessment of internal characteristics such as belief in oneself and personal competency is consistent with the growing strengths-based approach in child welfare to promote and strengthen resilience among these youth.

In conclusion, the current study’s findings provided evidence for the reliability and validity of the RS-14 for use in adolescents in the child welfare system. The short 14-item version is brief and easy to administer and has the potential to assess the personal characteristics and capacity for successful adaptation and positive outcomes among child welfare populations. The instrument may be useful as a screener for practitioners or researchers to identify youth who may benefit from interventions that promote or enhance resilience, as well as a valuable measure for evaluating the effectiveness of these interventions.
